# Integrin-uPAR signaling leads to FRA-1 phosphorylation and enhanced breast cancer invasion

**DOI:** 10.1186/s13058-018-0936-8

**Published:** 2018-01-30

**Authors:** Matthew G. Annis, Veronique Ouellet, Jonathan P. Rennhack, Sylvain L’Esperance, Claudine Rancourt, Anne-Marie Mes-Masson, Eran R. Andrechek, Peter M. Siegel

**Affiliations:** 1 Goodman Cancer Research Centre, McGill University, Montréal, Québec Canada; 2 Departments of Biochemistry, McGill University, Montréal, Québec Canada; 3 Departments of Medicine, McGill University, Montréal, Québec Canada; 4 Departments of Anatomy and Cell Biology, McGill University, Montréal, Québec Canada; 5 Centre de Recherche du Centre Hospitalier de l’Université de Montréal (CRCHUM) and Institut du cancer de Montréal, Montreal, Canada; 6 Department of Physiology, Michigan State University, East Lansing, Michigan USA; 7 Département de Microbiologie et Infectiologie, Faculté de Médecine et des Sciences de la Santé, Université de Sherbrooke, Sherbrooke, Canada

**Keywords:** Breast cancer, Integrins, uPAR, FRA-1, Invasion

## Abstract

**Background:**

The Fos-related antigen 1 (FRA-1) transcription factor promotes tumor cell growth, invasion and metastasis. Phosphorylation of FRA-1 increases protein stability and function. We identify a novel signaling axis that leads to increased phosphorylation of FRA-1, increased extracellular matrix (ECM)-induced breast cancer cell invasion and is prognostic of poor outcome in patients with breast cancer.

**Methods:**

While characterizing five breast cancer cell lines derived from primary human breast tumors, we identified BRC-31 as a novel basal-like cell model that expresses elevated FRA-1 levels. We interrogated the functional contribution of FRA-1 and an upstream signaling axis in breast cancer cell invasion. We extended this analysis to determine the prognostic significance of this signaling axis in samples derived from patients with breast cancer.

**Results:**

BRC-31 cells display elevated focal adhesion kinase (FAK), SRC and extracellular signal-regulated (ERK2) phosphorylation relative to luminal breast cancer models. Inhibition of this signaling axis, with pharmacological inhibitors, reduces the phosphorylation and stabilization of FRA-1. Elevated integrin α_V_β_3_ and uPAR expression in these cells suggested that integrin receptors might activate this FAK-SRC-ERK2 signaling. Transient knockdown of urokinase/plasminogen activator urokinase receptor (uPAR) in basal-like breast cancer cells grown on vitronectin reduces FRA-1 phosphorylation and stabilization; and uPAR and FRA-1 are required for vitronectin-induced cell invasion. In clinical samples, a molecular component signature consisting of vitronectin-uPAR-uPA-FRA-1 predicts poor overall survival in patients with breast cancer and correlates with an FRA-1 transcriptional signature.

**Conclusions:**

We have identified a novel signaling axis that leads to phosphorylation and enhanced activity of FRA-1, a transcription factor that is emerging as an important modulator of breast cancer progression and metastasis.

**Electronic supplementary material:**

The online version of this article (10.1186/s13058-018-0936-8) contains supplementary material, which is available to authorized users.

## Background

The transcription factor Fos-related antigen 1 (FRA-1) influences tumor heterogeneity [1] and is an important driver of cancer cell stemness and resistance in breast cancer [2]. FRA-1 is a member of the AP-1 family of transcription factors that regulate cell proliferation, differentiation, apoptosis and other biological functions and is encoded by the *fosl1* gene (reviewed in [3, 4]). They function as heterodimers composed of one Fos (c-FOS, FOSB, FRA-1 or FRA-2) and one JUN (c-JUN, JUNB or JUND) family member. FRA-1 was originally shown to transform Rat1 fibroblasts [5] and has since been implicated in the invasiveness and progression of several cancers [6–8], with a prominent role in enhancing the malignant phenotypes of breast cancer cells [9–12]. FRA-1 is also a target of the mircoRNA miR34, which is frequently downregulated in metastatic breast cancer cell lines and primary breast tumors with lymph node metastases. Forced expression of miR34 impairs cellular invasion and the ability of breast cancer cells to metastasize [13].

In breast cancer, FRA-1 expression is associated with the transition from normal epithelium to hyperplasia/ductal carcinoma in situ (DCIS) [14–16] and elevated FRA-1 correlates with increasing grade in invasive ductal carcinoma [2, 16]. Correlation between FRA-1 expression and clinical outcomes is more controversial. One study failed to detect an association between FRA-1 protein expression and overall survival [16], while others identified positive correlation between FRA-1 gene expression and shorter time to distant metastasis [2, 17, 18]. A curated FRA-1 transcriptional signature, when applied to numerous gene expression data sets, showed positive correlation with shorter time to distant metastasis or relapse across breast cancer subtypes [9, 10]. More recently, high FRA-1 expression was shown to be correlated with shorter overall survival and higher rates of lung metastases in patients with estrogen receptor (ER)-positive disease but not ER-negative cancers [19].

FRA-1 exerts pro-tumor functions through the numerous transcriptional targets it regulates [10, 20]. FRA-1 targets influence tumor cell proliferation, invasion and metastasis including: plasminogen activator, urokinase/plasminogen activator urokinase receptor (*plau/plaur*) [10, 21], matrix metalloproteinase 1 (*mmp-1*) [22], matrix-metalloproteinase-9 (*mmp-9*) [12], chloride channel accessory 2 (*clca2*) [18], adenosine receptor A2B (*ador2b*) [10], AXL tyrosine kinase receptor (*axl*) [23] and microRNAs, such as miR-221/222 [24]. FRA-1-regulated genes have demonstrated promise as potential therapeutic targets in breast cancer, including AXL [25] and adenosine receptor A2B [10].

Similar to other members in the Fos family of transcription factors [26, 27], FRA-1 is phosphorylated. Two serine residues, S252 and S265, in the c-terminal DEST sequence are phosphorylated, leading to increased protein stabilization by protecting FRA-1 from proteosomal degradation [23, 28–31]. FRA-1 transcriptional activity is correlated with protein stability and phosphorylation status [32] and the c-terminal region of FRA-1 is required for its transforming activity [33]. Receptor tyrosine kinase signaling (including epidermal growth factor receptor (EGFR) and MET), via the ERK pathway, has been shown to mediate FRA-1 phosphorylation in numerous cancers [34, 35]. FRA-1 can also be phosphorylated by protein kinase C (PKC)θ and PKCα on additional serine residues in the c-terminal DEST domain, which is thought to synergize with ERK-mediated phosphorylation to stabilize FRA-1 [2, 11, 36]. AKT signaling has also been shown to regulate the activity of AP-1 complexes, including FRA-1/c-JUN heterodimers [35].

Here, we demonstrate that engagement of the extracellular matrix protein vitronectin (VN), via the integrin and urokinase/plasminogen activator urokinase receptors (uPARs), leads to activation of SRC and mitogen-activated protein (MAP) kinase (MAPK) signaling and ultimately enhanced FRA-1 phosphorylation and the induction of breast cancer invasion.

## Methods

### Cell lines and culture conditions

The BRC-17, BRC-31, BRC-32, BRC-36 and BRC-196 cell lines were cultured as previously described [37]. All other breast cancer cell lines were obtained from the American Type Culture Collection (ATCC) and cultured as previously described [38]. Where indicated, cells were grown on fibronectin (2 ug/cm^2^; Millipore, Billerica, MA, USA), vitronectin (40 or 400 ng/cm^2^ as indicated; Peprotech, QC, Canada) or laminin (2ug/cm^2^; Trevigen, Gaithersburg, MD, USA).

### Reagents and DNA constructs

Dasatinib (LC Laboratories, Woburn, MA, USA), trametinib/dabrafenib/selumetinib/sorafenib (Selleckchem, Houston, TX, USA) and PP2 (Calbiochem, Gibbstown, NJ, USA) were dissolved in dimethylsulfoxide (DMSO) (Bioshop Canada, Burlington, ON, Canada) and added to fresh medium at the indicated concentrations.

Ten nanomoles of siRNA duplex (*fosl1* Smart pool: L-004341-00 (GE Healthcare Dharmacon Inc, Lafayette, CO, USA), *plaur* [29] or Scrambled (sequences listed in Additional file 1: Table S1) was transfected into cells using RNAiMax according to the manufacturers protocol (Life Technologies Inc., Burlington, ON, Canada). For the rescue of FRA-1 expression, two *fosl1* small interfering RNAs (siRNAs) that target the 3’ UTR were used (Additional file 1: Table S1).

The cDNA for *fosl1* was purchased from GE Healthcare Bio-Sciences Company (Lafayette, CO, USA) and cloned into an expression vector to add an HA-tag to the N-terminus. Phospho-deficient and phospho-mimetic versions were created using Quick-change mutagenesis (Agilent Technologies, Santa Clara, CA, USA) following the manufacturer’s directions. Sequences for the oligonucleotides used to make these mutants are listed in Additional file 1: Table S1.

### Immunoblotting

Thirty micrograms of protein was separated by SDS-PAGE and transferred to polyvinylidene fluoride (PVDF) membranes (Millipore, Billerica, MA, USA), where it was subsequently immunoblotted using the following antibodies: p44/42 MAPK, phospho-p44/p42 MAPK T202/Y204, phospho-FRA-1 S265, phospho-SFK Y416, Phospho-FAK Y925, Phospho-FAK Y576, Phospho-FAK 397, N-Cadherin, AKT, phospho-AKT S473 (Cell signaling, Whitby, ON, Canada); Integrins α_5_, α_v_, β_1_, β_3_, ErbB-2, FRA-1 (Santa Cruz Biotechnology, Dallas, TX, USA); α-Tubulin (Sigma, Oakville, ON, Canada), E-Cadherin (BD Biosciences, Mississauga, ON, Canada), uPAR (R&D Systems, Minneapolis, MN, USA), vimentin (Dako Canada Inc, Burlington, ON Canada), ER (Santa Cruz Biotechnology, Dallas, TX, USA), PR (Santa Cruz Biotechnology, Dallas, TX, USA) and cytokeratin-8 (a kind gift from Dr. Normand Marceau, Université Laval). Blots were incubated with either horseradish-peroxidase (HRP)-conjugated secondary antibodies (Jackson ImmunoResearch Laboratories, Bar Harbour, ME, USA), developed with chemiluminescent HRP substrate (ThermoScientific Inc, Waltham, MA, USA) and exposed to autoradiography film (Harvard Apparatus, Saint-Laurent, QC, Canada) or IR dye secondary antibodies (Licor Inc, Lincoln, NE, USA) and developed with the Odyssey Imager (Licor Inc, Lincoln, NE, USA). Quantification was performed using the ImageLite Studio software (Licor Inc, Lincoln, NE, USA).

### Extracellular matrix (ECM) stimulation and gene expression analysis

For the siRNA-mediated knockdown of uPAR, 48 hours post-transfection with siRNA, cells were harvested with 2 mM NaEDTA in PBS, washed with serum-free medium and plated for 30 minutes on culture dishes that were left uncoated or coated with the appropriate ECM. For gene expression analysis, cells were grown on the indicated ECM-coated or uncoated dishes for 18 hours prior to RNA extraction. For the rescue of *plaur*/*fosl1* knockdowns, cells were first transfected with siRNA then, 24 hours later, transfected with the indicated expression plasmid: 24 hours later, these cells were plated for 18 hours on VN-coated dishes prior to RNA extraction. RNA was extracted using RNeasy kits (Qiagen Inc, Toronto, ON Canada) according to the manufacturer’s protocol and reverse transcribed using a high-capacity cDNA reverse transcription kit (ThermoScientific Inc, Waltham, MA, USA). Gene expression analysis was performed using the LightCycler 480 and associated software using Advanced Relative Gene Expression Analysis (Roche Diagnostics, Laval, Quebec, Canada). The sequences used for qPCR primers are listed in Additional file 1: Table S1.

### Invasion assays

Cell invasion assays were performed as previously described [39] with the addition, where indicated, of vitronectin (24 ng/ml) within the Matrigel matrix alone or within both the Matrigel matrix and coated on the bottom surface of the Boyden chamber at 400 ng/cm^2^. In the latter assay, cells invade towards serum free-medium.

### In vivo tumor growth and establishment of explant cultures

The fourth mammary fat pad of ten athymic nude mice was injected with 1 × 10^6^ BRC cells (n = 10 mice/cohort). Tumor growth was monitored by weekly caliper measurements and volume calculated according to the formula:

$$ \left(\uppi \mathrm{LxW}\hat{\kern0.333em }2\right)/6 $$

where L refers to the length and W to the width of the tumor. After 9 weeks of growth, tumors were removed, digested with collagenase B and Dispase I (Roche Diagnostics, Laval, Quebec, Canada) and then incubated with cell growth medium to establish tumor explants.

### RNA preparation and microarray analysis

Total RNA was extracted using TRIzol reagent (Gibco/BRL, Life Technologies, Inc, Grand Island, NY, USA) according to the manufacturer's protocol. The quality of RNA was assessed using a 2100 Bioanalyzer with the RNA 6000 Nano LabChip kit (Agilent Technologies, Mississauga, ON, Canada) according to the manufacturer's protocol.

Microarray hybridization experiments were performed at McGill University and the Genome Quebec Innovation Center (Montreal, QC, Canada) using the HG-U133A GeneChip arrays. This chip allows the analysis of approximately 18,400 transcripts and variants, including 14,500 well-characterized human genes, composed of more than 22,000 probe sets. Protocols are available at the Affymetrix Web site (http://www.affymetrix.com/; Affymetrix, Santa Clara, CA, USA). Methods for labeling and hybridization of RNA were previously described [40].

### Gene expression statistical analysis

Raw microarray expression data from Neve et al., 2006 was downloaded from (http://www.ebi.ac.uk/arrayexpress/experiments/E-TABM-157/) and combined with the data from the five BRC cell lines and together they were pre-processed and clustered using GeneSpring software (V7.3, Agilent Technologies). Pre-processing included first robust multiarray averaging (RMA) normalization, then genes with expression below 0.01 were forced to meet this threshold, per-chip normalization was performed to the 50^th^ percentile, and per-gene normalization to the median. Data are presented as a log ratio, log2. Clustering was performed using the 305 gene classifier as previously described [38] using average linkage with similar branches merged and bootstrapping. The microarray data for the BRC cell lines can be accessed through the Gene Expression Omnibus (GEO) repository [GEO:GSE69915].

### Generation of the FRA-1 transcriptional activity signature

A publically available dataset ([GSE:46440] [18]) was used to generate an FRA-1 transcriptional activity signature. Significance analysis of microarrays (SAM) [41] was used to identify genes with 1.24-fold increase in control-transfected BT549 cells relative to BT549 cells transfected with an siRNA pool targeting *fosl1*. To apply the gene signature, the average of the signature genes was first calculated from RMA-normalized gene expression data and subsequently mean-normalized to obtain a value between 0 (low) and 1 (high). The signature was then validated on human breast cancer cell lines with either high or low FRA-1 phosphorylation (Fig. 7b) and only the top 65 genes within this signature were used for survival analysis (Fig. 7c). The number of available patient samples for analysis of overall survival (OS), relapse-free survival (RFS) and distant metastasis-free survival (DMFS) analyses was 3955, 1747 and 1402, respectively (Fig. 7f-h).

### Breast cancer dataset analysis

To assess potential clinical associations between FRA-1 levels, FRA-1 transcriptional activity, and molecular signaling components capable of activating FRA-1, the kmplot.com dataset [42] was used. Patients were split into high and low expression groups based upon the top quartile of patients versus the remainder of patients. Biased arrays were excluded from the analysis. No other filters were applied to the patient population before Kaplan-Meier analysis.

### Statistical analysis

GraphPad Prism 7.0 was used for statistical analysis. For sample sizes of three, the Shapiro-Wilk test was used to determine the normality of the data. For samples of eight or more the D'Agostino test was used to determine the normality of the data. Unless otherwise stated in the figure legends Student’s *t* test was used determine statistical significance. Supplemental methods and figure legends can be found in Additional file 2: Document 1: Supplemental Figure legends and Methods.

## Results

### Novel breast cancer lines derived from primary human breast tumors are representative of the intrinsic subtypes

**Fig. 1:**
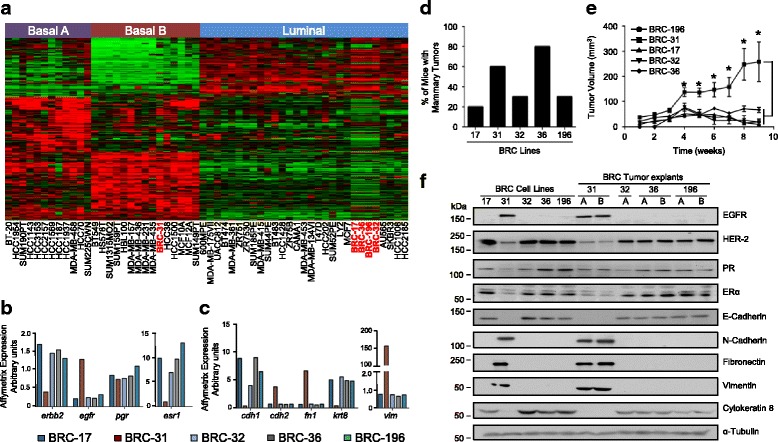
Molecular characterization of BRC cell lines established from primary human breast tumors. **a** Clustering of BRC explants with 51 established human breast cancer cell lines using a 305-gene subtype prediction signature. BRC cell populations are highlighted in red and the intrinsic subtype is indicated as basal A, basal B or luminal. **b** Gene expression data from the Affymetrix microarray for specific genes including: *erbb2*, epidermal growth factor receptor (*egfr*), progesterone receptor (*pr*) and estrogen receptor (*esr1*). **c** Gene expression data from the Affymetrix microarray for epithelial and mesenchymal markers including: E-cadherin (*cdh1*), N-cadherin (*cdh2*)*,* fibronectin (*fn1*)*,* cytokeratin 8 (*krt8*) *and* vimentin (*vim*)*.* **d** The percentage of mice with mammary tumors 9 weeks following mammary fat pad injection. **e** Tumor growth of BRC cell lines injected into the mammary fat pad of athymic mice. Mean tumor size for each cell line is plotted each week and the error bars represent the SEM. Statistical comparison is between BRC-31 and all other BRC-cell lines, *P < 0.01 (**f**) Immunoblot analysis of the original five breast cancer cell lines and their corresponding tumor explants (**a** or **b**) from four of the five cell lines. Representative blots from one of three independent sets of lysates are shown. α-Tubulin served as a loading control

We have examined a set of breast cancer cell lines that have been isolated directly from primary tumors of breast cancer patients [37]. We first characterized these explants by gene expression analysis and performed unsupervised clustering using a 305 gene signature [38] to classify human breast cancer cell lines as either luminal, basal A or basal B. Four of the five primary breast cancer explants (BRC-17, 32, 36 and 196) clustered with other luminal cell populations, whereas one explant (BRC-31) clustered closely with basal B cell lines (Fig. 1a). We specifically examined the expression of genes within the Affymetrix dataset that are characteristic of each intrinsic subtype including the estrogen receptor (*esr1*) and progesterone receptor (*pgr)* (luminal subtype), the ErbB2 receptor tyrosine kinase (*erbb2)* (human epidermal growth factor receptor 2 (HER2)^+^ subtype) or the epidermal growth factor receptor (*egrf*) (basal subtype). Luminal breast cancer cells (BRC-17, 32, 36 and 196) expressed higher levels of the *esr1* and *erbb2* relative to the BRC-31 cell population (Fig. 1b). Conversely, the BRC-31 explant exhibited high expression of *egfr* relative to the other explant populations, which is characteristic of 50% of basal breast tumors [43] (Fig. 1b). To further validate the basal nature of BRC-31 cells, we examined the expression of epithelial (E-cadherin (*cdh1*), cytokeratin-8 (*krt8*)) and mesenchymal (N-cadherin (*cdh2*), fibronectin (*fn1*) and vimentin (*vim*)) markers. BRC-31 breast cancer cells expressed high levels of *cdh2*, *fn1* and *vim* and low levels of *cdh1* and *krt-8* compared to BRC-17, 32, 36 and 196 cells (Fig. 1c). These data reinforce the classification of BRC-17, 32, 36 and 196 breast cancer cells as representative of the luminal subtype and the BRC-31 cell population as representative of the basal subtype.

When injected into the mammary fat pads of athymic mice, all five human explant cell lines were tumorigenic; however, only two cells lines (BRC-31 and BRC-36) demonstrated a reproducible ability to establish primary tumors (>60% incidence) and maintain sustained growth (Fig. 1d, e). No metastatic lesions were observed in lung tissue collected from tumor-bearing mice (data not shown). To verify phenotypic stability of these tumors following growth in the mammary fat pad of mice, we established explants from tumor-bearing mice (explant A or B). Immunoblot analyses revealed that BRC-31 breast cancer cells retained expression of EGFR, N-cadherin, fibronectin (FN) and vimentin at higher levels relative to BRC-32, 36 and 196 breast cancer cells (Fig. 1f). Conversely, BRC-31 cells exhibited low levels of Her2, estrogen receptor-α (ERα), E-cadherin and cytokeratin-8 relative to BRC-32, 36 and 196 cells (Fig. 1f). These data confirm the basal (BRC-31) and luminal nature (BRC-17, 32, 36 and 196) of these model systems and validate the Affymetrix gene expression profiles (Fig. 1a-c). While the incidence of tumor formation was similar, the BRC-36 cell line, but not the BRC-31 cell line, exhibited enhanced primary tumor growth in mice implanted with estrogen pellets (data not shown). Thus, we have characterized five novel breast cancer cell lines, four that represent ER-positive luminal breast tumors and one that represents basal breast tumors.

### Signaling via ERK2 leads to constitutive FRA-1 phosphorylation in basal B breast tumor cell lines

**Fig. 2:**
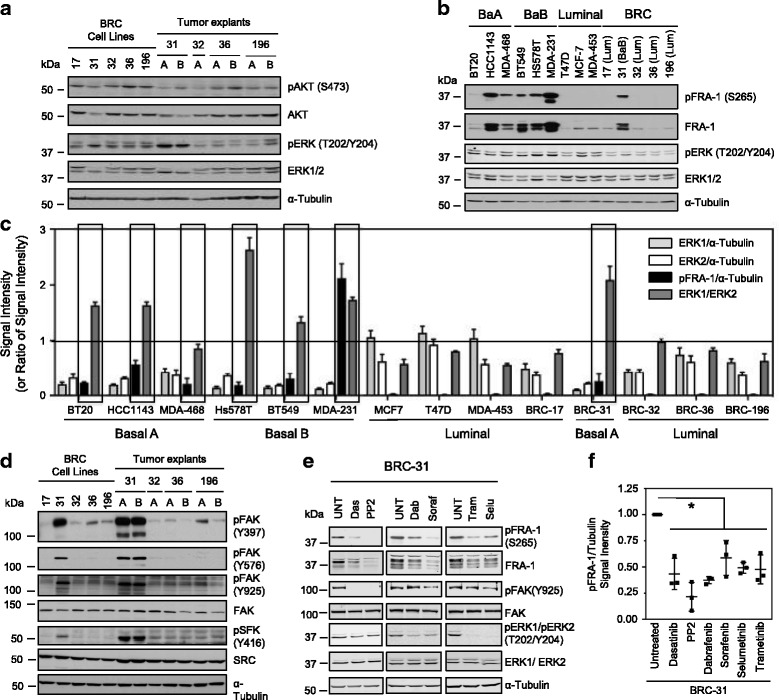
Phosphorylation of extracellular signal-related kinase 2 (ERK2) correlates with FRA-1 Ser265 phosphorylation in basal breast cancer cell lines. **a** Immunoblot analyses for AKT and ERK phosphorylation in five breast cancer cell lines and tumor explants (**a** or **b**) from the indicated cell populations. **b** Immunoblot analyses for Fos-related antigen 1 (FRA-1), phosphorylated FRA-1 (Ser265), pERK and ERK in a panel of human breast cancer cells (basal A, basal B and luminal) and BRC cell lines. **c** Quantification of ERK1 (ERK1/α-Tubulin) and ERK2 (ERK2/α-Tubulin) proteins normalized to α-Tubulin or presented as a ratio of ERK2 expression divided by ERK1 expression (ERK2/ERK1). Phosphorylated FRA-1 levels presented normalized to α-tubulin (pFRA-1/α-Tubulin). The average expression from three independent lysates is presented and error bars represent the standard error of the mean. **d** Immunoblot analyses for FAK and SRC phosphorylation in BRC cell lines and explants. **e** Immunoblot analyses of FRA-1 phosphorylation in BRC-31 breast cancer cells incubated with vehicle alone (UNT) or treated with SRC family kinase (SFK) inhibitors (25 nM Dasatinib; 6.6 μM PP2), rapidly accelerated fibrosarcoma (RAF) inhibitors (3 μM sorafenib; 3 μM dabrafenib) or mitogen-activated protein kinases (MEK) inhibitors (1 nM trametinib; 1 μM selumetinib) for 24 hours. **f** The level of phosphorylation of FRA-1 was quantified relative to the loading control α-Tubulin. The data from three independent lysates are plotted normalized to the dimethylsulfoxide DMSO vehicle control, *P < 0.02. Blots (**a**, **b**, **d**, **e**) are representative of at least three independent sets of lysates and α-Tubulin serves as a loading control. FAK, focal adhesion kinase

We next characterized the signaling pathways that were active in the BRC series of cell lines and derived tumor explants. We assessed the activation of the PI3-kinase pathway by examining AKT phosphorylation and the MAPK pathway by detecting ERK1/ERK2 phosphorylation. While the degree of AKT expression and phosphorylation was not noticeably different in the BRC cell lines and explants, we noted that the pattern of ERK1/2 phosphorylation, and to some extent expression of total ERK1/2, was clearly divergent between basal and luminal BRC cell lines. Specifically, we observed that the BRC-31 cell line and both tumor explants exhibited prominent p42 ERK2 expression and phosphorylation relative to the luminal BRC breast cancer cells (Fig. 2a). Previous observations have linked ERK2-dependent signaling to an epithelial-to-mesenchymal transition (EMT) that relied on phosphorylation and stabilization of FRA-1, a component of the AP-1 transcription factor family [31, 44]. Interestingly, the Fos-like antigen 1 (*fosl1)* gene, which encodes FRA-1, is overexpressed in basal breast cancer cell lines when compared to luminal breast cancer cells, with an overabundance specifically in basal B breast cancer cells (Additional file 3: Figure S1A).

The observation that BRC-31 breast cancer cells exhibit preferential ERK2 activation and express markers of an EMT transition (E-cadherin and cytokeratin-8 low; N-cadherin, FN and vimentin high) prompted us to examine the phosphorylation status of FRA-1. In the BRC panel, FRA-1 expression was uniquely elevated in the BRC-31 cells (Fig. 2b). FRA-1 is phosphorylated on serine residues 252 and 265, which leads to protein stabilization [23, 28–31]. In a panel of established human breast cancer cell lines (Fig. 2b) representing the luminal-like and basal-like subtypes we observed that, similar to previous reports [18], FRA-1 expression and phosphorylation of serine-265 is elevated in basal breast cancer cell lines (Fig. 2b). While ERK1 and ERK2 expression (protein, Fig. 2c; RNA, Additional file 3: Figure S1B) is variable across this panel of cell lines with no positive correlation with FRA-1 phosphorylation, the ratio of ERK2/ERK1 expression is associated with phosphorylation of FRA-1 (pFRA-1) (Fig. 2c, Spearman correlation value of 0.675 *P* = 0.01; RNA, *mapk1*/*mapk3* vs *fosl1*, Additional file 3: Figure S1A, Spearman correlation value of 0.451 *P* = 0.0004). These observations demonstrate that basal-like breast cancer cell lines possess elevated ERK2 expression relative to ERK1 and exhibit increased FRA-1 phosphorylation.

### A signaling axis involving FAK and SRC leads to FRA-1 phosphorylation in breast cancer cells

Given the elevated expression of EGFR in these cells (Fig. 1f), we anticipated that ERK activation occurred downstream of EGFR. While stimulation of BRC-31 cells with EGF led to an increase in pFRA-1/FRA-1 levels, inhibition of the EGFR with small molecule kinase inhibitors (AG1478 or gefitinib) did not alter ERK phosphorylation or basal pFRA1 status in BRC-31 cells (Additional file 4: Figure S2A, B). To identify the signaling pathways responsible for basal pFRA-1 levels in BRC-31 cells, we extended our characterization of the BRC panel of cell lines and examined FAK and SRC phosphorylation. We observed elevated phosphorylation on the FAK auto-phosphorylation site Y397 and on sites that are phosphorylated by SRC (Y576 and Y925) specifically in the basal BRC-31 cell line (Fig. 2d). Consistent with increased SRC activity we also observed increased SRC phosphorylation on Y416 in the BRC-31 cell line. This observation is of interest considering recent data implicating SRC in the phosphorylation of FRA-1 [45]. To determine if the SRC family kinase (SFK)-rapidly accelerated fibrosarcoma (RAF)-MEK pathway led to FRA-1 phosphorylation we treated cells with multiple small molecule kinase inhibitors that individually target each component of this pathway to identify optimal inhibitor concentrations, thus reducing the possibility of off-target effects. Inhibiting the activity of each protein kinase in this pathway reduced the level of FRA-1 phosphorylation relative to vehicle (Fig. 2e, f). These observations link SFK-RAF-MEK signaling activity to S265 phosphorylation on FRA-1.

### Extracellular matrix components engage integrin receptors for FRA-1 activation

**Fig. 3:**
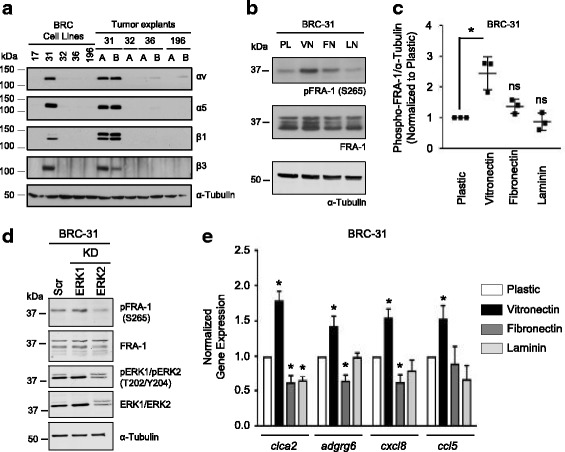
Vitronectin stimulates Fos-related antigen 1 (FRA-1) phosphorylation and FRA-1 transcriptional targets. **a** Immunoblot analyses of selected integrins in BRC cell lines and mammary tumor explants. **b** Immunoblot analyses of FRA-1 phosphorylation in BRC-31 cells plated on uncoated (PL), or dishes pre-coated with vitronectin (VN), fibronectin (FN) or laminin (LN). **c** The level of phosphorylation of FRA-1 was quantified relative to the loading control α-Tubulin. The data from three independent lysates are plotted normalized to the level of phosphorylation on uncoated dishes, *P < 0.01. **d** Immunoblot analyses of BRC-31 cells transfected with siRNAs against mitogen-activated protein kinase (*mapk1*) (extracellular signal-related kinase 2 (ERK2) knockdown (KD)), *mapk3* (ERK1 KD) or scrambled control (Scr), which were subsequently plated on vitronectin-coated cell culture dishes for 30 minutes. **e** Gene expression analysis of FRA-1 regulated transcriptional targets, chloride channel accessory 2 (*clca2*), adhesion G protein-coupled receptor G6 (*adgrg6*), C-X-C motif chemokine ligand 8 (*cxcl8*) and C-C motif chemokine ligand 5 (*ccl5*) following plating on uncoated or extracellular matrix (ECM)-coated cell culture dishes. Data are normalized to the expression from cells plated on uncoated dishes. Error bars are the standard error of the mean. *P < 0.03*.* Blots (**a**, **b**, **d**) are representative of at least three independent sets of lysates and α-Tubulin serves as a loading control

Given that EGFR inhibitors failed to diminish pFRA-1 levels, we reasoned that additional upstream receptors were responsible for SFK activation and ultimately FRA-1 phosphorylation. SFKs can be activated downstream of integrin engagement; thus, we assessed the expression levels of several integrin members. Interestingly, α_v_, α_5_, β_1_ and β_3_ integrin subunits were uniquely upregulated in BRC-31 breast cancer cells relative to the luminal BRC cell lines (Fig. 3a). These observations argue that integrin receptor-mediated FAK and SRC activation may represent a new mechanism leading to FRA-1 phosphorylation. Distinct integrin receptors bind to specific components of the ECM. For example, α_5_β_1_ receptors bind FN, α_V_β_3_ integrin receptors bind VN and α_2_β_1_ integrin receptors bind to laminin (LN). To determine which of these integrin heterodimers was responsible for increased FRA-1 phosphorylation, BRC-31 basal breast cancer cells were plated on VN, FN or LN. Only VN led to a significant increase in FRA-1 phosphorylation in BRC-31 breast cancer cells (Fig. 3b, c), which occurred prior to significant cell spreading on the ECM (Additional file 5: Figure S3A, B). To determine if VN-induced FRA-1 phosphorylation was mediated through ERK1/2, cells were transfected with siRNAs directed against either *mapk3* (ERK1), *mapk1* (ERK2) or scrambled control (Scrambled) and plated on VN-coated dishes (Fig. 3d). Only knockdown of ERK2, but not ERK1, diminished VN-induced FRA-1 phosphorylation (Fig. 3d).

Using available gene expression data from BT549 breast cancer cells transfected with siRNAs targeting *fosl1* [18] we generated an FRA-1 transcriptional signature composed of genes that are positively regulated by FRA-1 (Table 1). Using four of the top regulated genes from this list, we assessed whether activation of FRA-1 phosphorylation correlated with increased FRA-1 transcriptional activity. Consistent with previous reports linking FRA-1 transcriptional activity with phosphorylation [32], only cells plated on VN, but not FN or LN, exhibited a significant increase in FRA-1 regulated transcriptional targets (Fig. 3e).

**Table 1:** Genes highly expressed in BT549 breast cancer cells transfected with control siRNAs versus those treated with siRNAs targeting *FOSL1*

Symbol	Fold change	Symbol	Fold Change	Symbol	Fold change	Symbol	Fold change
*CLCA2*	6.489408	*C9orf41*	1.941214	*CPEB2*	1.544052	*TRIM16*	1.398898
*HPD*	4.227806	*KCTD14*	1.934207	*CPOX*	1.540511	*PSMB9*	1.398608
*ADGRG6*	3.283171	*GPR180*	1.929216	*ABI3BP*	1.531165	*PSMB9*	1.398608
*CXCL8*	3.158306	*ADAMTS3*	1.891931	*PTPRJ*	1.519587	*PSMB9*	1.398608
*CCL5*	3.085428	*ADAMTS1*	1.873739	*BMP4*	1.516994	*P4HA2*	1.389607
*C3*	3.012233	*NCRNA00052*	1.844217	*CXCL5*	1.516906	*LCLAT1*	1.388891
*VDR*	2.858543	*GRAMD3*	1.84082	*SGK196*	1.511883	*TRIM16L/ TRIM16*	1.377317
*INHBA*	2.727904	*EPHA4*	1.818525	*RAB9A*	1.505303	*RASA1*	1.371372
*KCNIP1*	2.696911	*BGN*	1.788637	*CDH2*	1.496587	*APOL6*	1.366417
*RGS5*	2.689024	*PDHX*	1.788554	*ERMP1*	1.489955	*SFRP2*	1.361497
*MX1*	2.581775	*SCN9A*	1.782825	*TULP3*	1.486287	*C1S*	1.358756
*BIRC3*	2.462052	*CCDC80*	1.776896	*ERAP1*	1.483427	*TGFBI*	1.35872
*C10orf54*	2.424571	*SH2D4A*	1.771079	*DDAH1*	1.481686	*AKIRIN1*	1.356119
*HBEGF*	2.396249	*SDPR*	1.764147	*POLR3G*	1.474358	*C3orf64*	1.355547
*IL1RAP*	2.340875	*TMEM2*	1.75761	*BAIAP2L1*	1.473152	*CTGF*	1.349245
*C5orf23*	2.322198	*CDH10*	1.751409	*BDNF*	1.472014	*HERPUD1*	1.348205
*KRT7*	2.308516	*ARHGDIB*	1.734891	*CRIM1*	1.468304	*GNAO1*	1.342846
*KRT8*	2.295598	*S100A8*	1.726534	*NMT2*	1.461901	*CCNJ*	1.342341
*GDF15*	2.278942	*VLDLR*	1.725248	*IER3*	1.46147	*PDP1*	1.342242
*INHBE*	2.212239	*IGFBP3*	1.678756	*HAS2*	1.453521	*HPSE*	1.341562
*IL7R*	2.186975	*ENPP1*	1.666838	*TNFSF15*	1.446719	*DICER1*	1.333887
*SRGN*	2.186939	*SEC24A*	1.659026	*FGF2*	1.441159	*RAG1AP1*	1.329645
*PLEKHA6*	2.17981	*DKK1*	1.657053	*FXYD7*	1.435982	*OTUD4*	1.312512
*IFI30*	2.144604	*ARSJ*	1.651702	*C21orf63*	1.434311	*RDH10*	1.30927
*KRT18 // MIR622*	2.13365	*TAP1*	1.635529	*GLIPR1*	1.433928	*EIF2B2*	1.30774
*APLN*	2.098161	*TAP1*	1.635529	*PRKAG2*	1.430065	*FSTL1*	1.306469
*NFE2L3*	2.093664	*TAP1*	1.635529	*C6orf145*	1.428615	*FAM98A*	1.297051
*GFRA1*	2.082099	*EDN1*	1.623509	*RCN1*	1.426605	*UHMK1*	1.296799
*HHIP*	2.060469	*USP18*	1.610502	*PGM2L1*	1.425401	*PRLR*	1.294388
*KRT18*	2.013096	*MTMR9*	1.59415	*GPX7*	1.419859	*KATNAL1*	1.284154
*IL6*	2.004191	*FGF5*	1.587693	*SLC19A3*	1.418788	*EBNA1BP2*	1.279577
*CXCL6*	1.997875	*JUB*	1.585937	*LAYN*	1.41537	*GYPC*	1.278302
*LRRN1*	1.989077	*P2RX5*	1.582798	*PIK3R1*	1.414605	*HLA-B*	1.270145
*TPD52L1*	1.969705	*EFEMP1*	1.580236	*SLC39A8*	1.409781	*TXNRD1*	1.25945
*CD14*	1.953506	*CCL2*	1.571968	*RAB32*	1.406182	*SLC35B1*	1.250224
						*OBFC2A*	1.246498

**Fig. 4:**
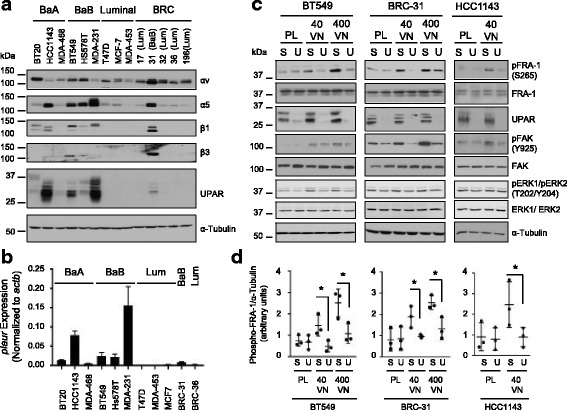
Vitronectin (VN)-stimulated Fos-related antigen 1 (FRA-1) phosphorylation requires urokinase/plasminogen activator urokinase receptor (uPAR) expression. **a** Expression of selected integrin subunits and uPAR in a panel of human breast cancer cell lines. **b** Quantitative PCR analysis of *plaur* expression in selected human breast cancer and BRC cell lines. Data presented are the mean expression from three independent RNA extractions and *plaur* expression is normalized to *actb*. Error bars represent the standard error of the mean. BaA basal A subtype; BaB basal B subtype; Lum, luminal subtype. **c** Immunoblot analyses of BT549, BRC-31 and HCC1143 cells transfected with siRNAs against *plaur* (U) or scrambled control (S), which were subsequently plated on plastic (PL), a low (40 ng/cm^2^) or high (400 ng/cm^2^) concentration of VN for 30 minutes. Representative blots from one of three independent experiments are shown. FAK, focal adhesion kinase; ERK, extracellular signal-related kinase. **d** The level of phosphorylation of FRA-1 was quantified and expressed as a ratio to the loading control α-Tubulin. The data from three independent lysates are plotted and the error bars represent the standard deviation between samples. *P ≤ 0.05

Urokinase plasminogen activator receptor (uPAR) is a known regulator of VN signaling in conjunction with integrin receptors [46, 47] and is a known transcriptional target of FRA-1 [48]. We speculated that uPAR could engage with specific integrin receptors to induce FRA-1 phosphorylation, which in turn maintains uPAR expression. Of the integrin receptors expressed in BRC-31 cells, α_5,_ β_1_ and β_3_ were also elevated in established basal breast cancer cells (Fig. 4a). There was also strong correlation between high levels of *plaur* (uPAR) expression and the basal subtype (Fig. 4a, b and Additional file 6: Figure S4). We asked whether reduction of uPAR expression in basal-like breast cancer cells would affect ECM-induced signaling and FRA-1 phosphorylation. When BT549, BRC-31 or HCC1143 cells were plated on plastic, knockdown of uPAR expression using siRNA-mediated approaches did not alter FRA-1 phosphorylation when compared with control-transfected cells (Fig. 4c, d). When plated on VN, BT549, BRC-31 and HCC1143 cells harboring control siRNAs, displayed an increase in FRA-1 phosphorylation. In contrast, when breast cancer cells with diminished uPAR expression were plated on increasing concentrations of VN, we observed a decrease in FRA-1 phosphorylation (Fig. 4c, d). FAK activation, measured by Y925 phosphorylation, was diminished with uPAR knockdown (Fig. 4c); however, we only detected a modest decrease in ERK phosphorylation despite the requirement for ERK2 expression for the phosphorylation of FRA-1 in these cells (Fig. 3c). Taken together, the data argue that VN engages an integrin/uPAR complex to induce downstream SRC/FAK signaling that ultimately leads to FRA-1 phosphorylation.

**Fig. 5:**
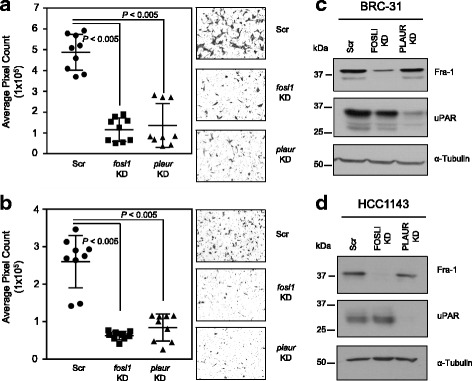
Vitronectin-induced breast cancer invasion requires urokinase/plasminogen activator urokinase receptor (uPAR) and Fos-related antigen 1 (FRA-1) expression. BRC-31 (**a**) or HCC1143 (**b**) breast cancer cells were plated in Boyden chambers in which the bottom surface was coated with vitronectin and the upper surface of the chamber coated with Matrigel mixed with vitronectin. Cells were allowed to invade towards serum-free medium for 24 hours. Quantification of breast cancer cell invasion and representative images are shown. Data from nine independent experiments plotted with the error bars representing the standard deviation. *P* values are as indicated. Immunoblot analyses of protein lysates from BRC-31 (**c**) or HCC1143 (**d**) cells transfected with scrambled (Scr), *fosl1* (*fosl1* knockdown (KD)) or *plaur* (*plaur* KD) siRNAs. α-Tubulin served as a loading control and representative blots from one of nine independent sets of lysates are shown

FRA-1 increases the invasive properties of breast cancer cells [12]. We postulated that mixing VN with Matrigel would further enhance BRC-31 invasion, as this would lead to increased integrin engagement and enhanced FRA-1 activity. To test this, we coated the bottom surface of the Boyden chamber with VN, mixed VN together with Matrigel in the chamber, and allowed the cells to invade towards serum-free medium. In the absence of VN, BRC-31 and HCC1143 cells did not invade through the Matrigel (data not shown), consistent with our previous observation that LN (the major component of Matrigel) failed to stimulate FRA-1 phosphorylation. In the presence of VN, BRC-31 and HCC1143 cells invaded through the Matrigel/VN mix (Fig. 5a, b, Scr); however, siRNA-mediated knockdown (KD) of FRA-1 (Fig. 5c, d) suppressed VN-induced BRC-31 and HCC1143 cellular invasion (Fig. 5a, b, *fosl1* KD). Consistent with a role for uPAR in stimulating the engagement of VN with integrins, siRNA-mediated knockdown of uPAR (Fig. 5c, d) reduced the invasive properties of BRC-31 and HCC1143 cells (Fig. 5a, b, *plaur* KD). Knockdown of FRA-1 or uPAR had no effect on cell proliferation over the duration of this assay (Additional file 7: Figure S5A-D). These data demonstrate that uPAR and FRA-1 are required for VN-induced cellular invasion.

### Phosphorylation of FRA-1 is required for transcriptional activity

**Fig. 6:**
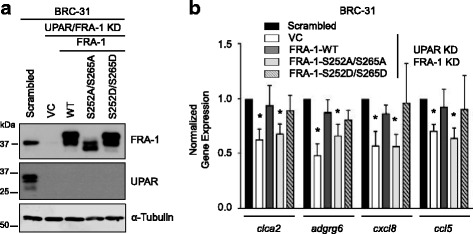
Phosphorylation of Fos-related antigen 1 (FRA-1) is required for transcriptional activity. **a** Immunoblots from BRC-31 cells transfected with siRNA to *plaur* and *fosl1* (UPAR KD/fFRA-1 KD) or scrambled control (Scrambled) were subsequently transfected with either empty vector (VC), FRA-1 wild-type (WT), FRA-1-S252AS265A (S252AS265A) or FRA-1-S252DS265D (S252DS265D) expression vectors. **b** Gene expression analysis of FRA-1 regulated transcriptional targets, chloride channel accessory 2 (*clca2*), adhesion G protein-coupled receptor G6 (*adgrg6*), C-X-C motif chemokine ligand 8 (*cxcl8*) and C-C motif chemokine ligand 5 (*ccl5*). Expression values are normalized to the scrambled control. Error bars represent the standard error of the mean. *P < 0.02

To determine if phosphorylation was required for FRA-1 activity, we knocked down the expression of both uPAR and FRA-1 with siRNA in BRC-31 cells (Fig. 6a) resulting in reduced expression of FRA-1 transcriptional targets relative to a non-targeting control (Fig. 6b), confirming the dependence, in part, on FRA-1 for the transcription of these targets. To rescue knockdown of endogenous FRA-1, we overexpressed either wild-type FRA-1, a phospho-deficient FRA-1 mutant (S252AS265A) or a phospho-mimetic FRA-1 mutant (S252DS265D) (Fig. 6a). Expression of wild-type FRA-1 or the phospho-mimetic mutant of FRA-1 restored expression of FRA-1 regulated transcriptional targets (Fig. 6b). In contrast, BRC-31 cells expressing the phospho-deficient mutant of FRA-1 displayed similar levels of these FRA-1 transcriptional targets to BRC-31 cells harboring the vector control (Fig. 6b).

### uPA, uPaR and FRA-1 are frequently co-expressed in human breast cancers

We analyzed representative breast cancer cells lines for expression of uPA, the ligand for uPAR, and observed that uPA mRNA expression and secretion was highest in basal breast cancer cells (Additional file 8: Figure S6A, B). We next wished to determine if this signaling axis was also upregulated in patients with breast cancer. As a first step, we examined tumor lysates from five breast cancer patient-derived xenografts (PDX) for the expression of uPAR, uPA and phosphorylated FRA-1 [49, 50]. Two of five PDX samples had elevated uPAR expression and detectable FRA-1 phosphorylation (Additional file 8: Figure S6C). The PDX sample showing the highest phospho-FRA-1 levels exhibited the highest uPA expression in tumor lysates (Additional file 8: Figure S6D).

**Fig. 7:**
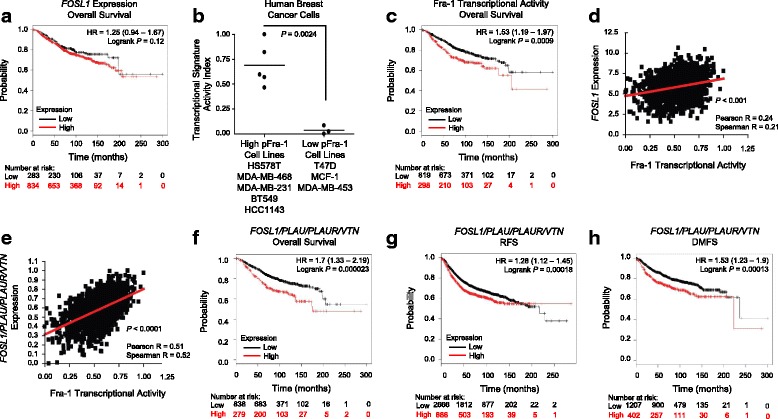
A molecular components signature of *plau/plaur/vtn/fosl1* is prognostic in human breast cancer. **a** Kaplan meier analysis of overall survival in patients with breast cancer separated by high or low *fosl1* expression. **b** Activity analysis of a Fos-related antigen 1 (FRA-1) transcriptional signature in breast cells with elevated phosphorylated FRA-1 (High pFRA-1 cell lines) or low levels of FRA-1 phosphorylation (Low pFRA-1 Cell lines). The activity index indicates the probability that a given breast cancer cell line will be positive for the FRA-1 signature. **c** Kaplan-Meier analysis in breast cancer patients using an FRA-1 transcriptional activity signature composed of the top 65 genes regulated by FRA-1 (see Table 1). Patients are divided into high (red) or low (black) transcriptional activity. **d** Correlation between *fosl1* expression and FRA-1 transcriptional activity signature. **e** Correlation between the FRA-1 transcriptional activity signature and molecular component signature composed of genes encoding FRA-1 *(fosl1)* and upstream signaling proteins (*plau/plaur/vtn*). **f** Kaplan-Meier analysis of breast cancer patients divided into high (red) and low (black) reveals that expression of a molecular component signature is prognostic for reduced overall survival (**g**), recurrence-free survival (RFS) and (**h**) distant metastasis-free survival (DMFS). KMplot.com dataset was used in all analysis apart from **b** where the Neve et al. dataset was used

We next assessed clinical correlation between *fosl1* expression, FRA-1 activity and breast cancer patient outcomes. Kaplan-Meier analysis of a human breast cancer dataset revealed that *fosl1* gene expression alone did not was not significantly prognostic of overall patient survival (Fig. 7a). To validate the robustness of the FRA-1 transcriptional signature (generated from available gene expression data [18] (Table 1)) as a surrogate readout of FRA-1 activity, we used it to segregate breast cancer cells in which FRA-1 phosphorylation status was previously established (Fig. 2b). Importantly, breast cancer cells characterized as high for the FRA-1 transcriptional signature were the same ones that displayed high FRA-1 phosphorylation (Fig. 7b) and the signature was prognostic of poorer overall survival in patients with breast cancer (Fig. 7c). Interestingly, we noted that the correlation between *fosl1* mRNA expression and presence of the FRA-1 expression transcriptional signature, while significant, was not very strong (Fig. 7d) consistent with *fosl1* expression alone not being prognostic in this dataset (Fig. 7a). This suggests FRA-1 expression alone does not translate to FRA-1 transcriptional activity. We speculated that breast tumors characterized by elevated expression for molecular components of the uPA/uPAR/VN/FRA-1 signaling axis might possess elevated FRA-1 transcriptional activity. Indeed, a molecular components signature (*plau/plaur/vtn/fosl1*) correlated well with FRA-1 transcriptional activity (Fig. 7e). Kaplan-Meier analysis of the same breast cancer dataset revealed that this molecular component signature was also prognostic in predicting poorer overall survival, recurrence-free survival and distant metastasis-free survival (Fig. 7f, g, h, respectively).

## Discussion

Phosphorylation provides a rapid mechanism to regulate FRA-1 transcriptional activity depending on the microenvironment encountered by the cell. Here we have described a vitronectin-stimulated pathway that leads to increased FRA-1 phosphorylation, transcriptional activity and invasion in basal-like breast cancer cells. Vitronectin is a soluble ECM protein that is found in the circulation and may provide a link between the components of the ECM, via collagen and heparin binding domains within vitronectin, and integrin binding-domain-containing proteins expressed on cells [51]. Vitronectin connects with assembled matrix proteins around wounds and thus may act as a bridge between the circulating tumor cells and areas of vascular damage. This leads to the intriguing possibility that cells engaging vitronectin may activate FRA-1 through increased phosphorylation and result in increased tumor cell extravasation. Indeed, vitronectin can stimulate increased tumor cell invasion [52, 53] and promote tumor growth [53] of breast cancer cells. Vitronectin may also play a role during intravasation as it can be detected in subendothelial regions and in small vessels surrounding tumor cells [54]. FRA-1 regulates the expression of multiple proteins involved in cell migration and invasion and it can also promote cell migration by suppressing RhoA activity; however, the exact mechanism has not yet been identified [55, 56].

The glycosyl phosphatidylinositol anchored membrane protein uPAR has been implicated in vitronectin-stimulated tumor cell migration and invasion [46, 47, 57]. As a membrane-associated protein, uPAR requires other membrane receptors including G protein-coupled receptors (GPCRs), certain growth factor receptors and integrin complexes to facilitate intracellular signaling (recently reviewed [58]). The β_1_-integrin and β_3_-integrin subunits are frequently reported as important signaling partners for uPAR [59]. Both uPAR and β3-containing integrin receptors can bind vitronectin, with uPAR recognizing the SMB domain and the β3 integrin subunit the Arg-Gly-Asp sequence of vitronectin; however, it is unclear whether a ternary complex indeed forms between uPAR-VN- β_3_-integrin [60]. Integrin signaling may be enhanced by uPAR through a direct conformational change in the β_3_-integrin subunit [61] or uPAR may alter the membrane surface leading to integrin activation [62]. It was recently reported that cell spreading induced by uPAR-vitronectin is not dependent on β_3_-integrin signaling, but requires non-ligand dependent activation of β_1_-containing integrin receptors, which is mediated through changes in membrane tension [62]. It is noteworthy that only vitronectin, and not fibronectin or laminin, was able to strongly stimulate FRA-1 phosphorylation in BRC-31 cells. It remains to be determined what additional cellular components are required to initiate this signaling axis.

Binding of urokinase plasminogen activator (uPA) to uPAR can enhance the binding of vitronectin to uPAR due to the fact that uPA and vitronectin utilize mutually exclusive binding sites to simultaneously bind uPAR [63, 64]. Upon uPAR binding, uPA is activated and cleaves the zymogen plasminogen into the active protease, plasmin, ultimately leading to the degradation of ECM components [65]. uPAR is also a substrate for uPA and the presence of soluble uPAR (suPAR) fragments in the circulation of pre-operative patients with breast cancer is indicative of poor prognosis [66]. As our experimental system removes any secreted protein, we suspect that uPA is not required for vitronectin-stimulated FRA-1 phosphorylation; however, it remains to be determined whether uPA is required for the invasive phenotype.

The controversial relationship between FRA-1 expression and clinical outcome in patients with breast cancer may be, in part, due to the fact that expression levels of FRA-1 may not correlate with phosphorylation status and transcriptional activity. To address this possibility, an “‘FRA-1 classifier” was constructed by identifying genes from an “‘FRA-1 transcriptome” (differentially expressed genes in MDA-MB-231 LM2 cells harboring short hairpin RNAs (shRNAs) to FRA-1 versus vector controls cells) that also had prognostic significance in publicly available datasets [10]. The Desmet et al. FRA-1 classifier showed significant prognostic ability to identify distant metastasis across all subtypes with the exception of Her2+/ER- breast cancers. This curated FRA-1 classifier provides a useful readout of FRA-1 activity; however, the subset of genes regulated by FRA-1 likely differ significantly depending cellular context, epigenetic variation and microenvironment. Similar to the datasets examined by Desmet et al., our Kaplan-Meier analysis using *fosl1* expression alone did not have prognostic significance in the independent breast cancer dataset utilized in this study; however, a gene signature containing the molecular components of the novel signaling pathway we have delineated in the present study (*plau/plaur/vtn/fosl1*) was able to identify patients with breast cancer with poor overall survival, recurrence-free survival and distant metastasis-free survival. We speculate that tumors with these signaling components would possess elevated FRA-1 activity and therefore be more aggressive in nature. Until now, FRA-1 phosphorylation has been described downstream of receptor tyrosine kinase signaling, via ERK2 [23, 29–31], and via members of the PKC family [2, 11]. Our data uncover a new signaling pathway, downstream of vitronectin engagement of integrin complexes that is augmented through the uPA/uPAR axis, which ultimately engages SRC/RAF/MEK to mediate FRA-1 phosphorylation.

Many components of the vitronectin-uPAR-integrin signaling axis are transcriptionally regulated by FRA-1 [10, 21], suggesting the existence of a positive feedback loop that further enhances FRA-1 activity. Strategies to suppress the pro-metastatic effects of FRA-1 include targeting downstream transcriptional targets and suppressing their activity [9, 10, 25]. However, given that numerous transcriptional targets likely contribute to the observed FRA-1 effects on breast cancer invasion and metastasis, such an approach may prove ineffective. Here we have demonstrated that vitronectin, and not laminin or fibronectin, stimulates FRA-1 phosphorylation via an uPAR-dependent process, suggesting that targeting this specific upstream axis could prove efficacious [67]. Application of the recently characterized small molecule inhibitors of uPAR-integrin association [68] and antibodies [69] may be beneficial in suppressing this signaling axis and reducing tumor cell invasion and metastases.

## Conclusions

We have identified a vitronectin stimulated signaling axis that leads to phosphorylation and stabilization of FRA-1, which is associated with increased transcriptional activity and breast cancer invasion. Notably, components of this signaling axis, along with a transcriptional signature of FRA-1 activity, are associated with poor clinical outcomes in patients with breast cancer. These data highlight FRA-1 as a transcription factor important for promoting breast cancer progression and metastasis.

## Additional files

Additional file 1: Oligonucleotides utilized in this manuscript. (XLSX 47 kb)

Additional file 2: Document 1: Supplemental Figure legends and Methods. (DOCX 86 kb)

Additional file 3: **Figure S1.** Gene Expression of mapk1, mapk3 and fosl1 in human Breast Cancer Cell lines. (PPTX 1395 kb)

Additional file 4: **Figure S2.** EGFR inhibition is not sufficient to decrease phosphorylation on FRA-1. (PPTX 4508 kb)

Additional file 5: **Figure S3.** FRA-1 phosphorylation occurs prior to cell spreading. (PPTX 4024 kb)

Additional file 6: **Figure S4.** Gene Expression of plaur in human Breast Cancer Cell lines. (PPTX 1129 kb)

Additional file 7: **Figure S5.** Knockdown of plaur or fosl1 does not affect cell proliferation. (PPTX 515 kb)

Additional file 8: **Figure S6.** Basal-like breast cancer cell lines and patient-derived xenografts (PDXs) that possess elevated FRA-1 phosphorylation display high uPAR and uPA expression. (PPTX 1129 kb)

## Abbreviations

ADORA2B: Adenosine receptor A2b
AXL: Axl tyrosine kinase receptor
CDH1: E-Cadherin
CDH2: N-Cadherin
CLCA2: Chloride channel accessory 2
DMSO: Dimethylsulfoxide
EGFR: Epidermal growth factor receptor
EMT: Epithelial-to-mesenchymal transition
ERK: Extracellular signal-regulated kinase
ESR1: Estrogen receptor 1
FAK: Focal adhesion kinase
FN: Fibronectin
FRA-1: Fos-related antigen 1
HER2: Human epidermal growth factor receptor 2
KD: Knockdown
KRT8: Cytokeratin-8
LN: Laminin
MAP: Mitogen-activated protein
MAPK: Mitogen-activated protein kinase
MMP-1: Matrix metalloproteinase-1
MMP-9: Matrix metalloproteinase-9
PBS: Phosphate-buffered saline
PDX: Patient-derived xenograft
PKC: protein kinase C
PSR: Progesterone receptor
RAF: Rapidly accelerated fibrosarcoma
Scr: Scrambled
SFK: SRC family kinase
siRNA: small interfering RNA
UPA: Urokinase plasminogen activator
UPAR: Urokinase/plasminogen activator urokinase receptor
UTR: Untranslated region
VIM: Vimentin
VN: Vitronectin
