# Supplementary material for: Integrin-uPAR signaling leads to FRA-1 phosphorylation and enhanced breast cancer invasion
Source: Breast Cancer Res. 2018 Jan 30;20:9. doi: 10.1186/s13058-018-0936-8 (PMC5791353; doi:10.1186/s13058-018-0936-8)
Supplement: Supplementary file 2 — Document 1: Supplemental Figure legends and Methods. (DOCX 86 kb) [file 13058_2018_936_MOESM2_ESM.docx]

**Supplementary Material**

**Supplemental Methods**

**EGFR Inhibition and EGF stimulation**

AG1478 (Enzo Life Sciences, Farmingdale, NY, USA), Gefitinib (LC Laboratories, Woburn, MA, USA) were dissolved in dimethylsulfoxide (DMSO) (Bioshop Canada, Burlington, ON, Canada) and added to fresh media at the indicated concentrations to BRC-31 cells. For stimulations experiments, 1 ng/ml of recombinant EGF (Peprotech, Quebec, Canada) was added to serum free media for the indicated times.

**Cell Proliferation**

Cell proliferation was determined using a modified Crystal-Violet assay [1]. Briefly, cells were stained 0.5% Crystal-Violet then washed four times with PBS and the Crystal-violet extracted with 0.33% Acetic Acid. Absorbance was measured at 570nm.

**ECM Stimulation**

Cells were harvested with 2mM NaEDTA in PBS, washed with serum free media and plated for 5, 10, 20 or 30 minutes on culture dishes that were left uncoated or coated with the 400 ng/cm^2^ vitronectin. After incubation, plates were washed with PBS and lysed in TNE lysis buffer. Images were captured using a on BX-45 inverted-microscope (Olympus and Infinity Analyze software, Richmond Hill, ON Canada).

**ELISA**

uPA expression was measured by ELISA following the manufacturer’s directions (R&D Systems, Minneapolis, MN, USA) using either tumor cell lysates or conditioned media collected from breast cancer cell lines incubated with serum free media for 48 hours.

**Figure Legends**

**Fig. S1**. Gene Expression of *mapk1, mapk3* and *fols1* in human Breast Cancer Cell lines. Gene expression data from the Affymetrix microarray of (**A**) *fosl1* and a ratio of *mapk1/mapk3* and (**B**) *mapk1* and *mapk3* in breast cancer cell lines representing three subtypes: Basal A, Basal B and Luminal.

**Fig. S2.** EGFR inhibition is not sufficient to decrease phosphorylation on FRA-1. (**A**) Immunoblots of cell lysates derived from BRC-31 cells were treated with 1 ng/ml of EGF for the indicated times. (**B**) Immunoblots of cell lysates derived from BRC-31 cells treated with 0.2, 2, and 20 nM of AG1478, 0.2, 2 and 20 nM of Gefitinib or DMSO (vehicle control) for 24 hours.  Immunoblots were probed with the indicated antibodies, α-Tubulin served as a loading control.

**Fig. S3.** FRA-1 phosphorylation occurs prior to cell spreading. (**A**) Immunoblots of BRC-31 lysates following incubation on uncoated (Plastic) or Vitronectin coated (VN) cell culture dishes for the indicated time. α-Tubulin serves as a loading control. (**B)** Images of BRC-31 plated on either uncoated (Plastic) or vitronectin coated (Vitronectin). Scale bar indicates 50 µm.

**Fig. S4**. Gene Expression of *plaur* in human Breast Cancer Cell lines. Gene expression data from the Affymetrix microarray of *plaur* in breast cancer cell lines representing three subtypes: Basal A, Basal B and Luminal.

**Fig. S5.** Knockdown of *plaur* or *fosl1* does not affect cell proliferation. Immunoblots of cell lysates derived from BRC-31(**A**) or HCC1143 (**B**) transfected with siRNA scrambled control (SCR) or siRNA to *plaur* (*plaur* KD) or *fosl1* (*fosl1* KD). Cell lysates are from Day three of the proliferation assay and α-Tubulin serves as a loading control. Proliferation assay of BRC-31 (**C)** or HCC1143 (**D**) cells transfected with Scrambled controls (Scrambled), *fosl1* (*fosl1* KD) or *plaur* (*plaur* KD) siRNAs. The average of three experiments is plotted and normalized to Day 0. Error bars are the standard deviation.

**Fig. S6.** Basal-like breast cancer cell lines and patient-derived xenografts (PDXs) that possess elevated FRA-1 phosphorylation display high uPAR and uPA expression. (**A**) Normalized Affymetrix gene expression data for *plau* in human-derived breast cancer cell lines. (**B**) Conditioned media collected from the indicated cell lines was subjected to ELISA analysis to determine the concentration of uPA in the media. Data was normalized for cell plating variability by dividing the concentration of uPA in the media by the concentration of protein in the media. (**C**) Immunoblot analyses of uPAR, pFRA-1 and FRA-1 in triple negative breast cancer (TNBC) PDXs. Representative blots from one of three independent sets of lysates are shown. α-Tubulin served as a loading control. (**D**) ELISA of uPA expression in Breast PDX tumor lysates. Error bars represent the standard deviation between three samples.

**Table S1.** Oligonucleotides utilized in this manuscript

**References:**

1. Gillies RJ, Didier N, Denton M: **Determination of cell number in monolayer cultures**. *Analytical biochemistry* 1986, **159**(1):109-113.
