# Supplementary material for: Integrin-uPAR signaling leads to FRA-1 phosphorylation and enhanced breast cancer invasion
Source: Breast Cancer Res. 2018 Jan 30;20:9. doi: 10.1186/s13058-018-0936-8 (PMC5791353; doi:10.1186/s13058-018-0936-8)
Supplement: Supplementary file 4 — Figure S2. EGFR inhibition is not sufficient to decrease phosphorylation on FRA-1. (PPTX 4508 kb) [file 13058_2018_936_MOESM4_ESM.pptx]

## Slide 1
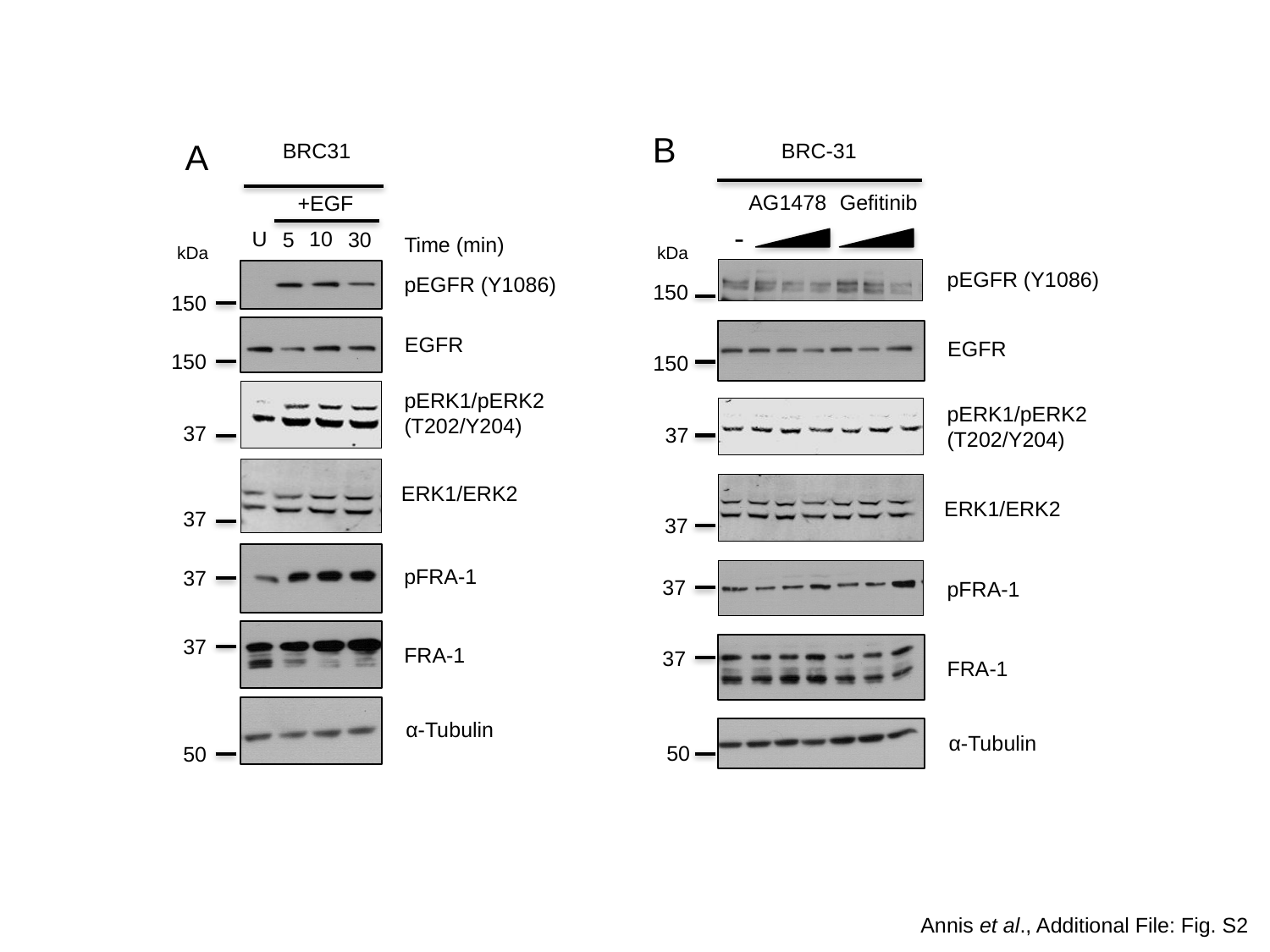

B
BRC-31
AG1478
Gefitinib
-
kDa
pEGFR (Y1086)
150
EGFR
150
pERK1/pERK2
(T202/Y204)
37
ERK1/ERK2
37
37
pFRA-1
37
FRA-1
α-Tubulin
50
A
BRC31
+EGF
U
10
30
5
Time (min)
kDa
pEGFR (Y1086)
150
EGFR
150
pERK1/pERK2
(T202/Y204)
37
ERK1/ERK2
37
pFRA-1
37
37
FRA-1
α-Tubulin
50
Annis et al., Additional File: Fig. S2
