# Supplementary material for: Integrin-uPAR signaling leads to FRA-1 phosphorylation and enhanced breast cancer invasion
Source: Breast Cancer Res. 2018 Jan 30;20:9. doi: 10.1186/s13058-018-0936-8 (PMC5791353; doi:10.1186/s13058-018-0936-8)
Supplement: Supplementary file 5 — Figure S3. FRA-1 phosphorylation occurs prior to cell spreading. (PPTX 4024 kb) [file 13058_2018_936_MOESM5_ESM.pptx]

## Slide 1
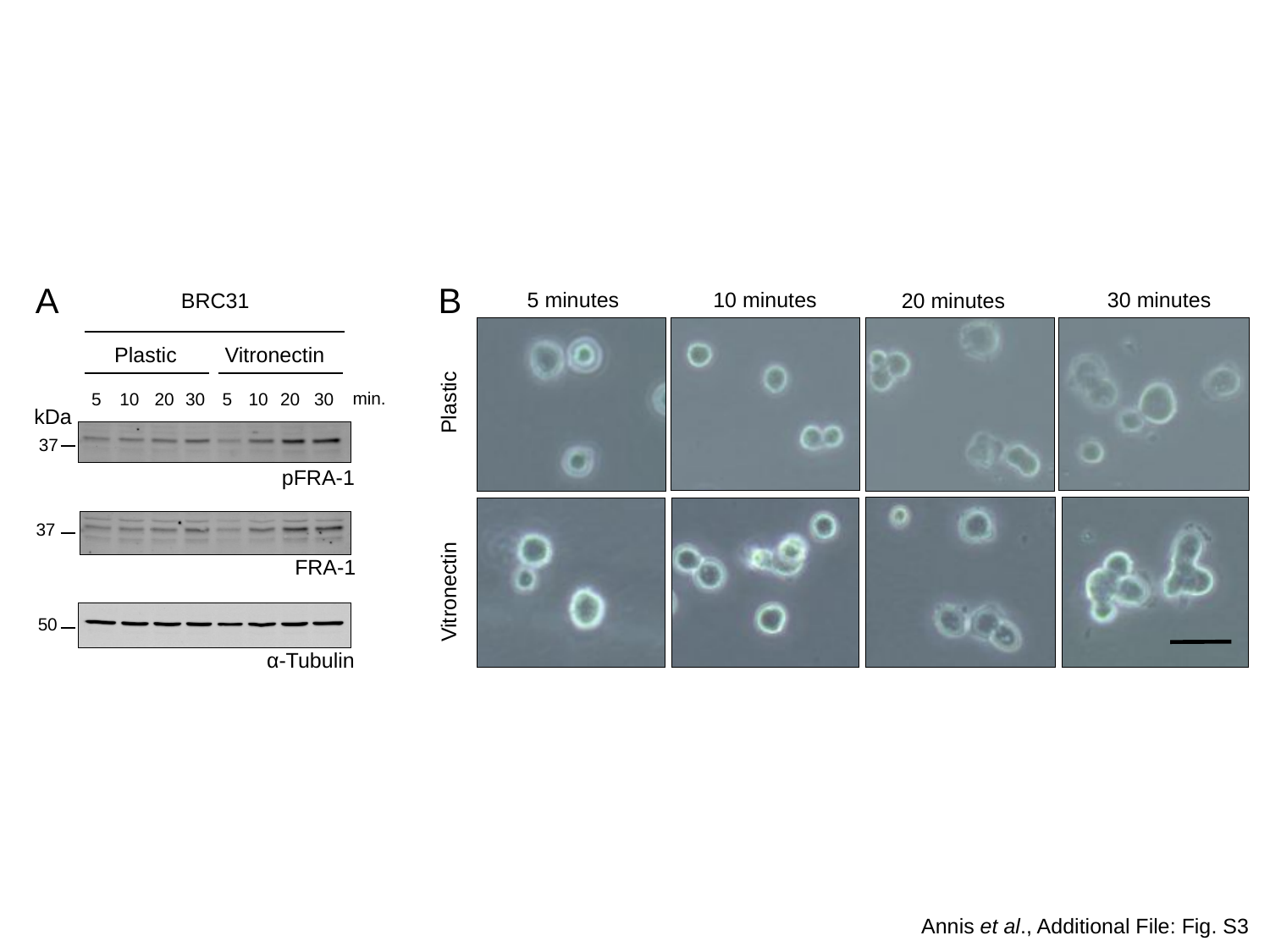

A
BRC31
Plastic
Vitronectin
min.
5
10
20
30
5
10
20
30
kDa
37
pFRA-1
37
FRA-1
50
α-Tubulin
B
5 minutes
10 minutes
30 minutes
20 minutes
Plastic
Vitronectin
Annis et al., Additional File: Fig. S3
