# Supplementary material for: Integrin-uPAR signaling leads to FRA-1 phosphorylation and enhanced breast cancer invasion
Source: Breast Cancer Res. 2018 Jan 30;20:9. doi: 10.1186/s13058-018-0936-8 (PMC5791353; doi:10.1186/s13058-018-0936-8)
Supplement: Supplementary file 6 — Figure S4. Gene Expression of plaur in human Breast Cancer Cell lines. (PPTX 1129 kb) [file 13058_2018_936_MOESM6_ESM.pptx]

## Slide 1
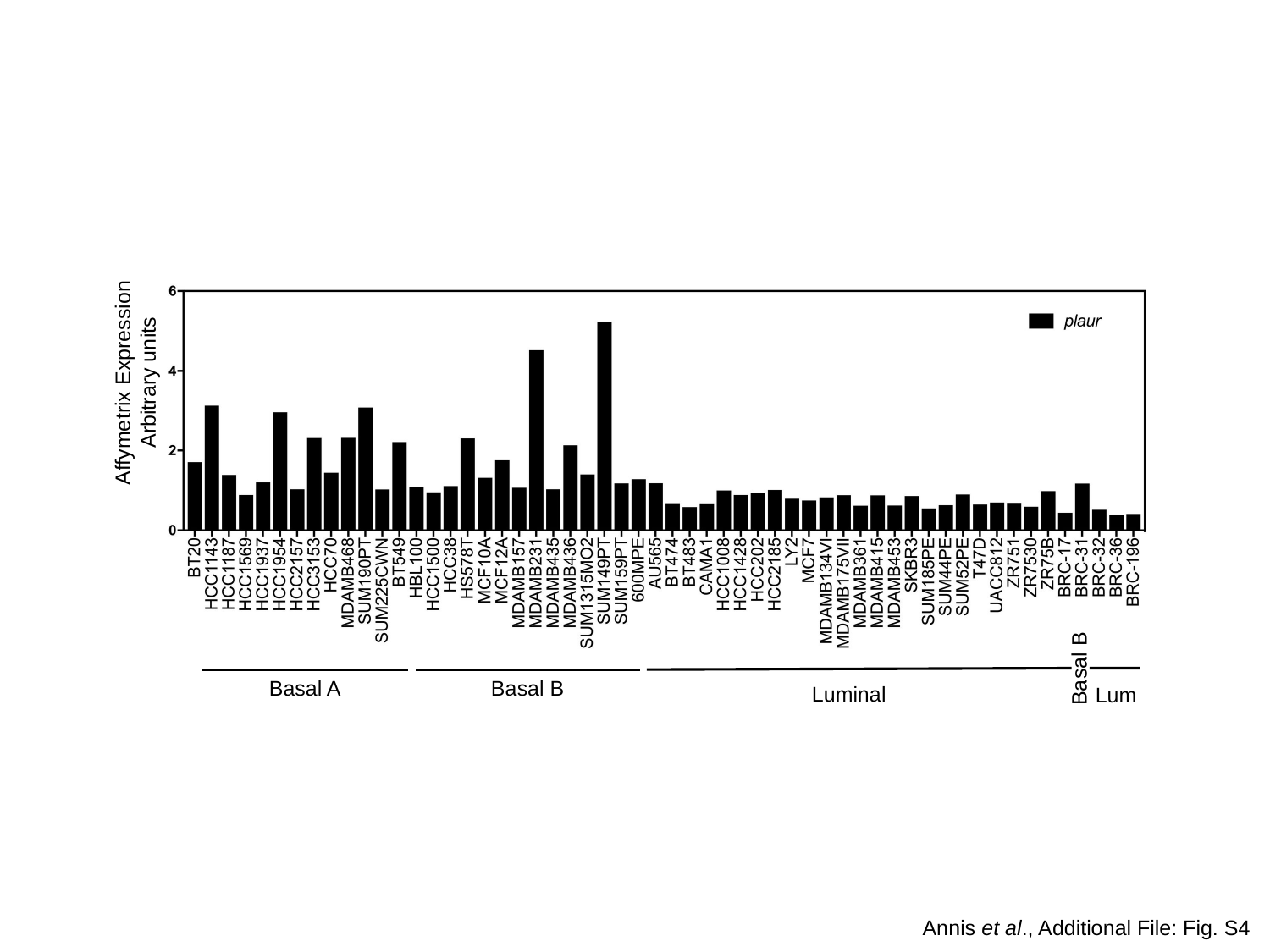

Affymetrix Expression
Arbitrary units
Basal B
Basal B
Basal A
Luminal
Lum
Annis et al., Additional File: Fig. S4
