# Supplementary material for: Integrin-uPAR signaling leads to FRA-1 phosphorylation and enhanced breast cancer invasion
Source: Breast Cancer Res. 2018 Jan 30;20:9. doi: 10.1186/s13058-018-0936-8 (PMC5791353; doi:10.1186/s13058-018-0936-8)
Supplement: Supplementary file 7 — Figure S5. Knockdown of plaur or fosl1 does not affect cell proliferation. (PPTX 515 kb) [file 13058_2018_936_MOESM7_ESM.pptx]

## Slide 1
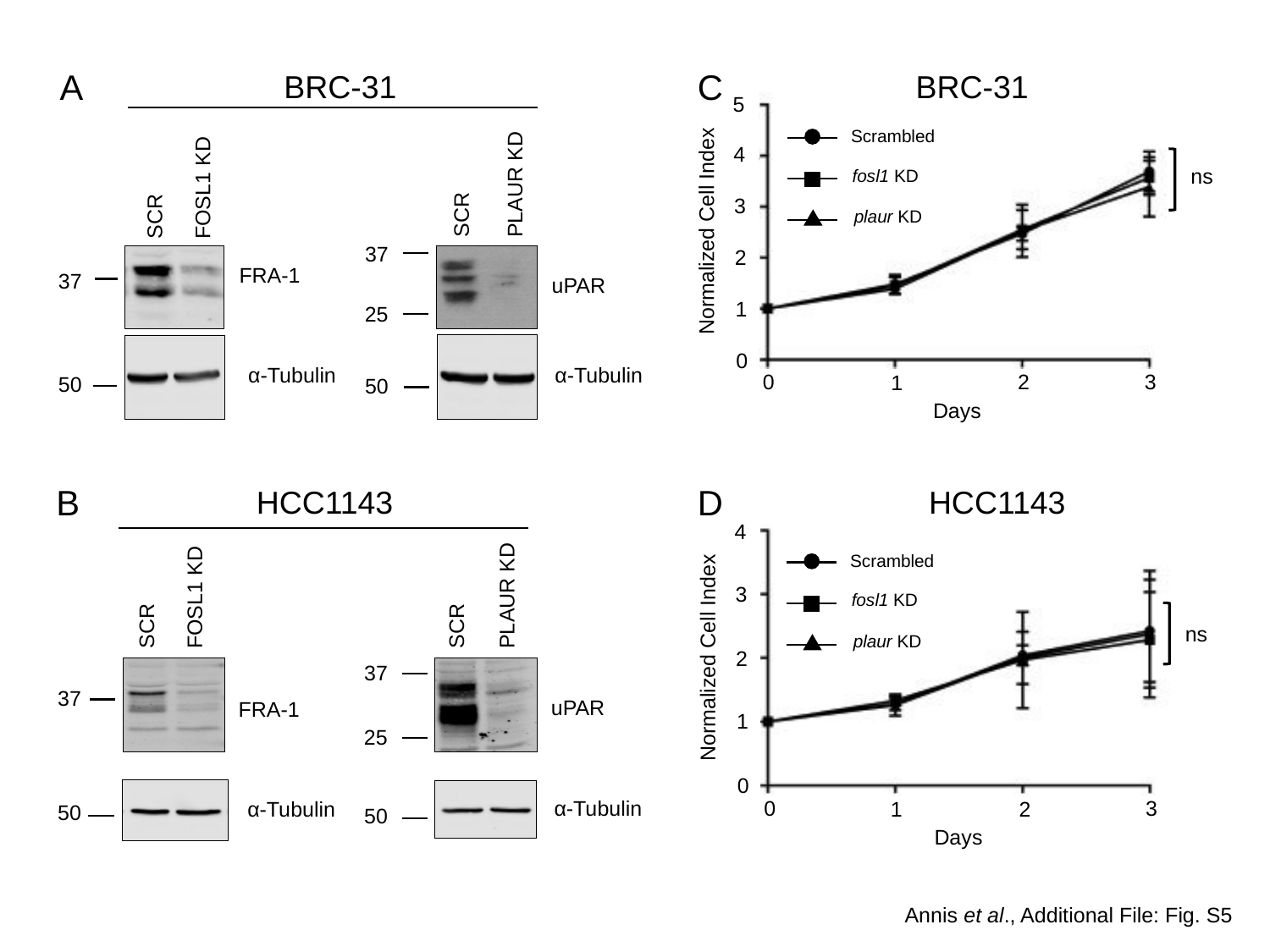

A
BRC-31
PLAUR KD
FOSL1 KD
SCR
SCR
FRA-1
37
uPAR
25
α-Tubulin
α-Tubulin
50
50
C
BRC-31
5
Scrambled
4
ns
fosl1 KD
3
plaur KD
Normalized Cell Index
2
1
0
3
0
2
1
Days
B
HCC1143
PLAUR KD
FOSL1 KD
SCR
SCR
37
uPAR
FRA-1
25
α-Tubulin
α-Tubulin
50
50
D
HCC1143
4
Scrambled
3
fosl1 KD
ns
plaur KD
Normalized Cell Index
2
1
0
3
0
2
1
Days
37
37
Annis et al., Additional File: Fig. S5
