# Supplementary material for: Integrin-uPAR signaling leads to FRA-1 phosphorylation and enhanced breast cancer invasion
Source: Breast Cancer Res. 2018 Jan 30;20:9. doi: 10.1186/s13058-018-0936-8 (PMC5791353; doi:10.1186/s13058-018-0936-8)
Supplement: Supplementary file 8 — Figure S6. Basal-like breast cancer cell lines and patient-derived xenografts (PDXs) that possess elevated FRA-1 phosphorylation display high uPAR and uPA expression. (PPTX 1129 kb) [file 13058_2018_936_MOESM8_ESM.pptx]

## Slide 1
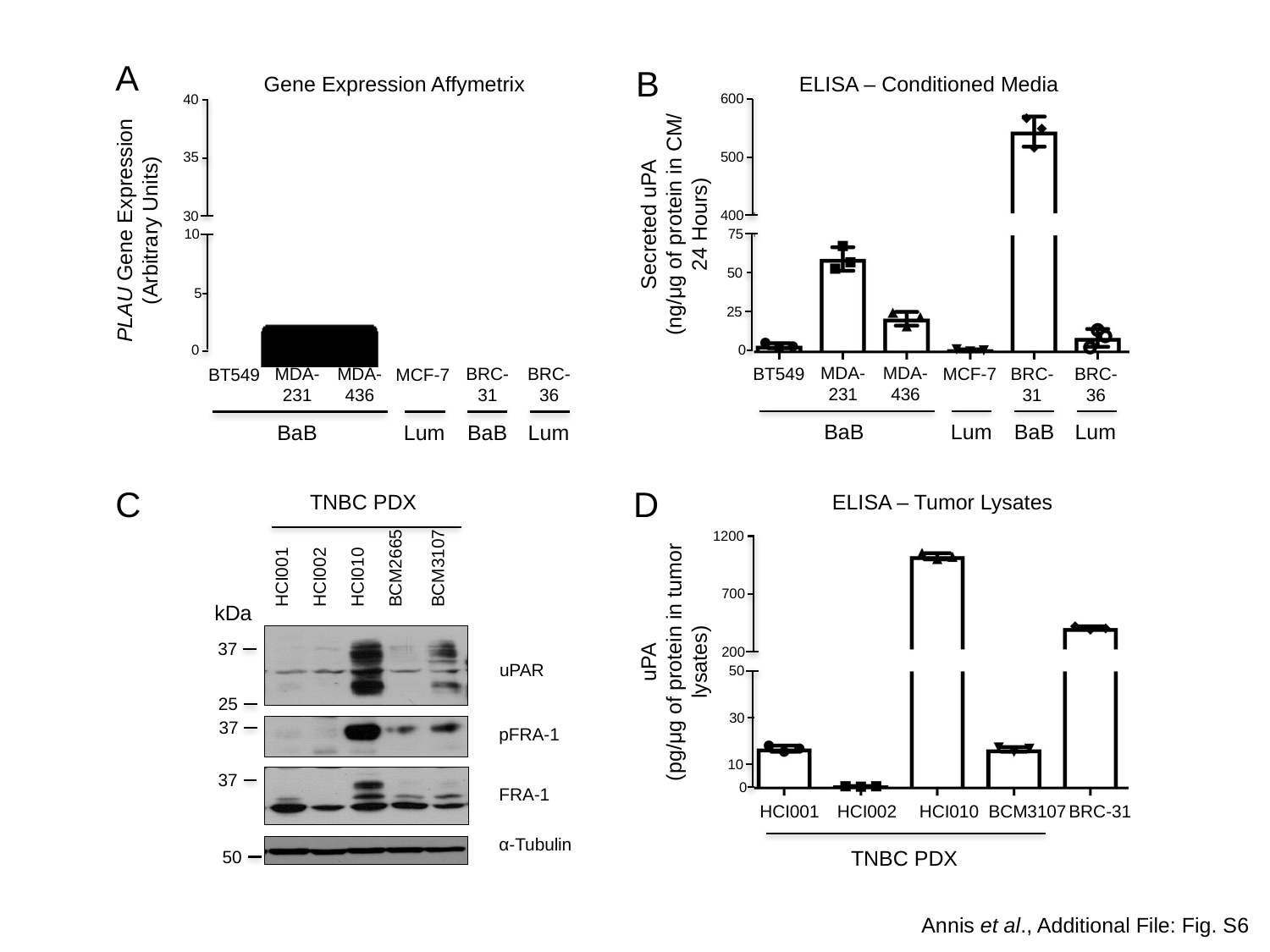

A
Gene Expression Affymetrix
40
35
30
5
0
10
PLAU Gene Expression
(Arbitrary Units)
MDA-
231
MDA-
436
BRC-
31
BRC-
36
MCF-7
BT549
BaB
Lum
BaB
Lum
B
ELISA – Conditioned Media
600
500
400
50
0
75
Secreted uPA
(ng/μg of protein in CM/
24 Hours)
25
MDA-
231
MDA-
436
BRC-
31
BRC-
36
MCF-7
BT549
BaB
Lum
BaB
Lum
C
TNBC PDX
BCM2665
BCM3107
HCI001
HCI002
HCI010
kDa
37
uPAR
25
37
pFRA-1
37
FRA-1
α-Tubulin
50
D
ELISA – Tumor Lysates
1200
700
200
30
0
50
uPA
(pg/μg of protein in tumor
lysates)
10
BCM3107
BRC-31
HCI001
HCI002
HCI010
TNBC PDX
Annis et al., Additional File: Fig. S6
